# Pilot Study of the Relationship between Deck Level and Journey Duration on Plasma Cortisol, Epinephrine and Norepinephrine Levels in Italian Heavy Pigs

**DOI:** 10.3390/ani10091578

**Published:** 2020-09-04

**Authors:** Giancarlo Bozzo, Barbara Padalino, Elisabetta Bonerba, Roberta Barrasso, Vincenzo Tufarelli, Martina Zappaterra, Edmondo Ceci

**Affiliations:** 1 Department of Veterinary Medicine, University of Bari, Strada Provinciale per Casamassima km 3, 70010 Valenzano, Italy; giancarlo.bozzo@uniba.it (G.B.); elisabetta.bonerba@uniba.it (E.B.); edmondo.ceci@uniba.it (E.C.); 2 Department of Agricultural and Food Sciences, University of Bologna, Viale Giuseppe Fanin 46, 40127 Bologna, Italy; barbara.padalino@unibo.it (B.P.); martina.zappaterra2@unibo.it (M.Z.); 3 Department of DETO-Section of Veterinary Science and Animal Production, University of Bari, Strada Provinciale per Casamassima km 3, 70010 Valenzano, Italy; vincenzo.tufarelli@uniba.it

**Keywords:** pigs, stress, transport, cortisol, catecholamines, deck level

## Abstract

**Simple Summary:**

Journey duration, position of the animal in the vehicle and time spent in the lairage facilities before slaughtering can negatively affect pig welfare. The effects of journey duration and deck level on hormonal parameters were examined in this pilot study during three commercial truckloads. Plasma cortisol, epinephrine and norepinephrine increased with longer transportation time. The levels of the tested hormones were higher in pigs transported for 11 h than for 6.5 h or only 3 h. The loading position (deck level) of the animals on the truck strongly affected only norepinephrine. While the effects of journey duration on plasma cortisol levels were expected and in line with the literature, our findings on plasma catecholamines are novel and may be useful to monitor transportation stress responses in pigs. These preliminary findings need to be confirmed by future studies.

**Abstract:**

The aim of this pilot study was to evaluate the relationship between journey duration, deck level and activation patterns of the hypothalamic-pituitary-adrenocortical axis (HPA) and sympathetic adrenal medullary system (SAM) in pigs. A total of 90 pigs were examined. The animals came from three different Italian farms associated with the same slaughterhouse located in Bari (Apulia region-Italy). A group of thirty animals was transported from Pordenone (11 h journey); a second group was transported from Terni (6.5 h journey); a third group was transported from Benevento (3 h journey). The animals were transported in the same vehicle, which complied with the structural characteristics indicated in the Council Regulation (EC) No. 1/2005. The truck was composed of a lorry and a trailer, each one divided into three decks. Only the animals transported in the trailer were tested for the study. Before transportation, blood samples were collected on each farm, at 6:00 a.m., from 30 pigs randomly selected out of 135 pigs ready to be transported. Blood samples were also collected during slaughter to evaluate plasma cortisol, epinephrine and norepinephrine, around 6:00 a.m. A journey duration of 11 h was associated with significantly higher plasma concentrations of stress hormones compared with shorter journeys. This increase was proportional to the journey duration, with the pigs travelling for 6.5 h displaying intermediate concentrations between those noticed after 3 h and 11 h journeys. The interaction between deck and journey distance was not significant on epinephrine, norepinephrine or cortisol levels collected at arrival. There was a significant effect of deck level on norepinephrine levels (*p* < 0.0001), a tendency to influence epinephrine levels (*p* = 0.073) but no effect on cortisol levels (*p* = 0.945). Overall, we observed that an 11 h-long journey seemed to impact negatively on pigs’ HPA-SAM activity, likely requiring the animals to spend more time in the lairage facilities to recover.

## 1. Introduction

Transport is a crucial and critical factor in modern pork production [1]. In the European Union, transport covers over 365 million livestock (poultry excluded), including 225 million pigs [2]. However, transport towards slaughterhouse represents a stressor for pigs, affecting their health and welfare and consequently their carcass quality [3]. During transport, pigs are subjected to many adverse situations including weather conditions [1]. Several studies focused on the influence of factors related to transport including loading density, handling treatment, trailer design, loading method, environmental and internal climate conditions, duration of the journey [4,5,6]. Regarding duration, journeys are divided into short (<8 h) and long journeys (>8 h) although the Member States may grant derogations for journeys not exceeding 12 h to reach the final place of destination [7]. Depending on the length of the journey, the vehicles must guarantee certain requirements that for short journeys include cleaning, flooring surface, sufficient lighting, adequate ventilation, partitions, suitable equipment for loading and unloading. For long journeys additional requirements are included, i.e., roof, floor and bedding, feed, water supply, ventilation and temperature monitoring, and a navigation system. In addition, regardless of journey duration, the position of the animals in the vehicle during transport can affect skin blemishes and meat quality [8,9]. Indeed, the truck deck affects animal stress levels in a highly variable way [10]. However, the impact of deck levels on stress indicators in pigs is still unclear.

There are two axes primarily involved in animal stress response: the sympathetic adrenal medullary system (SAM) and the hypothalamic-pituitary-adrenocortical axis (HPA) [11]. Activation of the sympathetic nervous system in emergencies involves the release of catecholamines (epinephrine and norepinephrine) from the adrenal medulla and of cortisol from the zona fasciculate of the adrenal cortex [12]. For stress evaluation, the quantification of cortisol (or its metabolites) constitutes an effective and precise indicator of the physiological state of an animal, as well as an index of its response to environmental conditions [13,14]. Consequently, measurement of circulating cortisol concentration is common in research to evaluate pre-slaughter stress [10].

Blood cortisol levels in pigs varied within studies, depending on the duration of the journey, the time spent in the lairage facilities and the deck levels where the pigs were transported [15,16,17]. Transport duration was significant for cortisol concentration and pigs transported for 6 h displayed a greater cortisol concentration than pigs transported for 3 h (284.1 vs. 263.4 ng/mL) [10]. After transport from a farm to the abattoir of only 15 min, the cortisol concentrations varied among the pigs from 3 to 248 nmol/L, with a mean of 58.36 nmol/L [18]. On the other hand, in a study where the average duration of transport to the slaughterhouse was 40 min [19], the cortisol values ranged from 363.3 to 417.9 nmol/L. The results of these studies have often been conflicting owing to differences in trailer design, deck level and journey duration. Moreover, there is no information on the plasma variations of epinephrine and norepinephrine in pigs after transport.

Based on the above-mentioned background, the aim of the present study was to evaluate the stress induced by transport of Italian heavy pigs destined for the dry-cured ham industry. These animals can reach a body weight between 150 and 180 kg at the end of their production cycle. We tested the hypothesis that the levels of stress indicators would be associated with truck decks and journey duration. Accordingly, we monitored the effects of journeys of different durations (3, 6.5 and 11 h) towards the same slaughterhouse on plasma cortisol, epinephrine and norepinephrine levels in pigs transported in three deck levels. Additionally, the possibility provided by Council Regulation (EC) No. 1/2005 to extend a short journey by an additional 4 h in a vehicle approved only for short journeys was investigated.

## 2. Materials and Methods

### 2.1. Ethical Statement

The experimental procedures were approved by the ethical committee of the Department of Veterinary Medicine at Bari (Italy) University (Protocol n. 933-III/13, 15 April 2020). The study was conducted after collecting owners’ consent, without affecting the routine of farms and slaughterhouses.

### 2.2. Sampling

This pilot study, resulting from a partnership between the Department of Veterinary Medicine at Bari University (Italy) and a slaughterhouse in Apulia region (Siciliani-Meat Processing Industry, Bari) was conducted during April and May 2019.

Three pig farms were selected as associated with Siciliani-Meat Processing Industry. For this reason, they were managed similarly, applying the same production disciplinary. In particular, during the trial, pigs were fed the same diet (ingredients composition: corn, barley, soybean meal, wheat middlings, wheat bran, molasses, soybean oil, vitamin-mineral premix, monocalcium phosphate, calcium carbonate, sodium chloride) formulated to meet the nutrient requirements for fattening pigs according to the National Research Council recommendations [20]. They were reared under similar conditions, in environmentally controlled (lighting and ventilation program) rooms with concrete floor and equipped with a self-feeder and a nipple waterer to allow pigs ad libitum access to feed and water throughout the fattening period. The space allocation in farms was 0.78 m^2^/pig with 10 pigs per pen. The pigs were also of the same sex (males) and genotype (Danish Duroc, Talent line). The day before transport, all animals had an average body weight of 150 kg and about 110 days of age. Pigs were fasted about 12 h before transport.

A total of 90 pigs from 3 farms (30 for each farm) were included in the study. Farm A (group A) was located in Pordenone (Friuli Venezia Giulia region, Italy), 888 km from the slaughterhouse, located in Bari (Apulia Region, Italy); farm B (group B) was located in Terni (Umbria region, Italy), 485 km far from the slaughterhouse; farm C (group C) was located in Benevento (Campania region, Italy), 222 km far from the slaughterhouse. Group A animals were transported on 5 April 2019 (888 km; journey time: 11 h; mean temperature: 11 °C; humidity: 89%), group B animals on 11 April 2019 (485 km; journey time: 6.5 h; mean temperature: 11 °C; humidity: 85%) and group C animals on 30 April 2019 (222 km; journey time: 3 h; mean temperature: 12 °C; humidity: 70%). The three journeys were performed on three different days, but in the same truck driven by the same driver.

On each journey, the truck was loaded with 135 pigs (i.e., the full load of the truck) and the decks were loaded sequentially, from the upper to the lower deck. The animals travelled by road in the same vehicle, which complied with the structural characteristics required for a journey of fewer than 8 h, which can be increased up to 12 h, as indicated in the Council Regulation (EC) No. 1/2005 [7]. In compliance with Council Regulation (EEC) No. 3820/85 of 20 December 1985 on the harmonisation of certain social legislation relating to road transport [21], the driver from Benevento did not make any stops from departure to arrival at the slaughterhouse whilst the driver from Terni made a single stop and the driver from Pordenone made two stops. Each stop was about 1 h long, recorded in the route plan of the truck and carried out in dedicated shaded areas, without unloading the animals.

**Figure 1:**
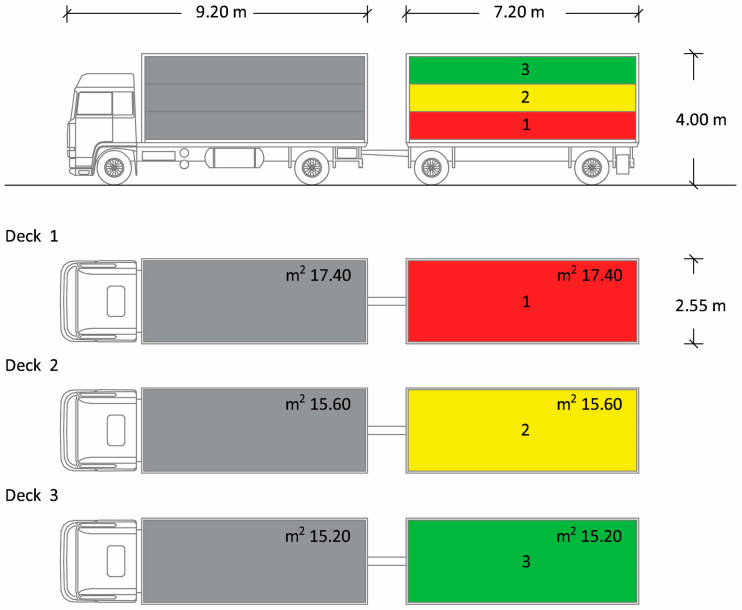
Schematic drawing of the truck used for pig transportation. The truck was composed of a lorry and a trailer and sectioned into 3 compartments: decks 1, 2 and 3. Only the animals travelling in the trailer were included in the study.

The truck was equipped by Carrozzeria Pezzaioli (Montichiari, Italy), composed of a lorry and a trailer, each one divided into three decks (Figure 1) containing 22–23 pigs/deck. The total loading capacity of the truck was about 20,000 kg (equivalent to 130–135 market-weight pigs). The lorry and the trailer body had the same dimensions: 7.20 m long × 2.55 m wide × 4.00 m high. The truck was stocked following the European livestock transport rule Council Regulation (EC) No. 1/2005 [7], according to which, to guarantee that all pigs must at least be able to lie down and stand up in their natural position, the loading density for pigs of around 100 kg should not exceed 235 kg/m^2^. For this purpose, in our study, the available space allowance was about 0.71 m^2^/pig, approximately 212 kg/m^2^, i.e., a density lower than that required by law. The vehicles were equipped with suitable drinking systems that always allowed the supply of water during the journey. Lorries and trailers had both natural and mechanical ventilation systems, consisting of six automatic fans. Ventilation systems were capable of maintaining, at any time during the journey, a range of temperatures from 5 to 30 °C within the means of transport, for all animals, with a ±5 °C tolerance, depending on the outside temperature. The nominal airflow capacity of the installed fans was greater than 60 m^3^/h/KN of payload and the system was capable of operating for at least 4 h, independently of the vehicle engine.

On each farm, the day before loading, blood samples (5 mL) were collected from all departing pigs by the farm veterinarian to check animal health. To avoid influences on the diurnal rhythm of the hormonal secretion, all samples in the three farms were carried out at the same hour (6:00 a.m.) and also the truck departures were scheduled to make sure that their arrival at the slaughterhouse was around 5:00 a.m. in order to perform the animal bleeding between 5:00 and 6:00 a.m. As this was an opportunistic study, we analysed the samples of 30 pigs randomly selected from the 135 pigs ready to be transported. Animals were restrained to obtain a blood sample from the ear vein through a needle (22 G) connected to the syringe. The technique used was a low-stress restraint method that did not require the use of a snare or excessive containment. In particular, the restraint of the animals was performed, without using tranquilisers or anaesthetics, keeping one pig at the time inside a restraining cage, while the farm veterinarian carried out the blood collection. Blood sampling from the ear causes lower levels of stress than jugular blood sampling because of reduced handling and containment [22]. After obtaining the blood sample, pressure was applied to the ear vein for several seconds to induce clotting. Blood samples were collected using vacutainer test tubes containing ethylenediaminotetracetic-acid (EDTA) as anticoagulant. Then, the samples were stored in ice-cold water for no longer than 60 min, avoiding freezing, before submitting to the laboratory used by the farm for the routine blood analysis. At the laboratory, blood tubes were centrifuged at 4 °C for 10 min at 2000× *g*; plasma was then transferred into 1.5-mL Eppendorf tubes and stored at −20 °C until analysis. These tubes were sent, in dry ice, to the Department of Veterinary Medicine at Bari University for testing.

At the slaughterhouse, the date and the duration of the journeys were recorded, and the routes taken by the vehicle were noted. Moreover, the official veterinarian carried out a checklist to double check whether the animal welfare conditions during transport were respected [7]. At the end of the official controls, all pigs were unloaded using an exit ramp equipped with a non-slip floor and fitted with side guards. The pigs were unloaded very quickly (within 10 min). The trailer was unloaded before the lorry and only the animals transported in the trailer were tested for the study; the pigs were marked with different colours depending on the truck deck in which each animal group had travelled (first/red, second/yellow and third/green). The decks were unloaded sequentially, from the lower to the upper deck and the pigs were immediately conducted inside the slaughterhouse for the stunning phase, without a resting period. The animals were slaughtered in the same order in which they were unloaded. Once the unloading was completed, each animal was immediately directed to the stunning area, consisting of a small corridor where the pig was bathed at head level and then introduced into a cage. Then, the pigs were subjected to the stunning phase by electro-narcosis (Gozlin Electronic stunner TEQ002, serial number: TEQHZV19, year of manufacture: 2019), applying the electrodes at the level of the animals’ heads, delivering a voltage of 250–300 V for 1–2 s [23]. After checking that stunning had correctly occurred, the exsanguination phase was performed. Subsequently, during exsanguination from the jugular vein, 30 pigs (10 per truck deck) were randomly selected and blood samples were collected between 5:00 and 6:00 a.m., without disturbing the normal routine of the slaughterhouse. These samples were handled in the same way as those collected in the three farms and stored at −20 °C for analysis.

### 2.3. Plasma Cortisol-Elisa Test

Plasma cortisol was determined as described in previous studies [24,25]. The cortisol ELISA immunoassay test (Porcine Cortisol ELISA Kit; My-Bio-Source, San Diego, CA, USA) was used following the manufacturer’s guidelines. Briefly, the optical density (OD) of each well was determined using a microplate reader (GloMax^®^ Discover Microplate Reader, Promega Corporation, Madison, WI, USA) with a detection wavelength filter of 450 nm and correction wavelength filters of 570 nm or 630 nm. The mean of the readings of duplicates for each standard and sample was calculated, and the average OD of the blank was subtracted. A standard curve was created using computer software capable of generating a four-parameter logistic (4-PL) curve-fit. The minimum detectable porcine cortisol (sensitivity) was up to 5 ng/mL. The detection range was 1000–15.6 ng/mL. No cross-reaction or interference between porcine cortisol and other factors were observed. Intra-assay precision was ≤8%, while inter-assay precision was ≤12%.

### 2.4. Plasma Catecholamine hPLC Test

The catecholamines plasma kit (ClinRep; Recipe Chemicals and Instruments GmbH, Munchen, Germany) was designed for the quantitative determination of catecholamines from plasma with High-Performance Liquid Chromatography (HPLC). HPLC with electrochemical detection was established as a reliable and sensitive method for the determination of catecholamines in plasma. The Clin-Cal Plasma Calibrator and the Clin-Chel Plasma Controls were lyophilised and needed to be reconstituted before use with deionised water. One millilitre of plasma was pipetted into the sample preparation column (directly onto the aluminium oxide suspension) and subsequently, 50 μL of internal standard was added. The column was closed, and it was shaken upside down for 10 min. The column was slightly tapped on to transfer these residues back to the bottom of the frit column. Then, the upper and lower caps of the sample preparation column were removed, the supernatant was aspirated (with a vacuum station) and the effluent was discarded. One millilitre of washing solution was transferred to the sample preparation column and then the washing solution was aspirated. The washing procedure was carried out three times and, after these steps, the effluent was discarded. The elution vial was plugged onto the sample preparation column and 120 μL of eluting reagent was pipetted into the column. Afterwards, it was mixed for 1 min on a vortex mixer. Subsequently, the eluate was centrifuged through the column into the elution vial (for 1 min at 1000× *g*), which may be used for subsequent sample injection in the auto-sampler. The HPLC pump flow rate was 1 mL/min. The analytical column was installed in the column heater at 25 °C. Auto-sampler injection volume: 40 μL HPLC System (prepared sample, calibrator or controls). Injection interval: 15 min. Electrochemical detector parameters: potential 500 mV; sensitivity 10 nA; filter setting 0.2 Hz.

The concentration of the analytes was calculated with the internal standard method via the peak areas. According to the internal standard method, each sample was spiked with a so-called “internal standard” before the sample preparation. The internal standard was similar to the analytes in terms of behaviour during sample preparation and chromatography. Hence, any losses during the sample preparation could be determined by calculating the recovery. The extrapolation to 100% recovery allowed the determination of the concentration of the unknown substances in the sample. Both norepinephrine and epinephrine had linearity of 15–2500 ng/L (lower limit of detection: 8 ng/L; lower limit of quantitation: 15 ng/L).

### 2.5. Statistical Analysis

Statistical analyses were performed in R environment [26]. First, normality of the data was checked using Shapiro test in the stats package. Whilst epinephrine levels were normally distributed, cortisol and norepinephrine levels were not, and thus were transformed using Box-Cox Power Transformation in car package [27] and rescaled with functions in scales package. Then, the normally distributed data were analysed using two-way ANOVA where the effects of time (T0 = before transport; T1 = after transport), farm of origin (Farm A, B, C), and the interaction between time and farm were tested on the levels of plasma cortisol, epinephrine and norepinephrine.

Furthermore, to evaluate the effect of the truck deck and journey duration, the data collected at arrival (T1) were analysed using two-way ANOVA with the journey distance (J1, J2, J3 corresponding to the farm of origin, A, B, C), the 3 deck levels (1, 2, 3) and the interaction between journey distance and deck as factors, and plasma cortisol, epinephrine and norepinephrine levels as outcomes. Tukey test was used as post-doc and data were expressed as Least Squares Mean (LSM) ± Standard Error (SE). The ANOVA and the LSM were performed with stats and emmeans packages. The significance threshold was set at or below 5%.

## 3. Results

There was a significant effect of time, farm of origin and their interaction on cortisol, epinephrine and norepinephrine levels (*p* < 0.0001 for all the factors) (Table 1). Transport was significant for the levels of stress hormones. An almost 30-fold increase in plasma norepinephrine concentrations was observed after transport. Similarly, epinephrine showed, on average, a 16-fold increase at T1 and cortisol concentrations tripled after transport. The farm of origin, i.e., the journey distance, was also significant for the amounts of stress hormones in the plasma of the transported pigs. Long-distance journey (from farm A, in Pordenone, 11 h-long journey) was associated with significantly higher plasma concentrations of the tested hormones compared with shorter journey distances (from Terni, farm B, 6.5 long journey and Benevento, farm C, 3 h-long journey). This increase was proportional to the journey distance, with the pigs travelling for 6.5 h displaying intermediate concentrations between those observed in animal travelling for 3 h and animals travelling for 11 h (Table 1).

## 4. Discussion

The pilot study compared plasma cortisol and catecholamine levels in pigs transported in three different deck levels during transport of different durations (short, 222 km/3 h; medium, 485 km/6.5 h; medium long 888 km/11 h). Overall, the findings of this pilot study supported the initial hypothesis that the plasma concentrations of the tested hormones were higher in pigs subjected to medium-long distance transport than in pigs subjected to a medium or short distance transport, as shown by the progressive increases of the levels of these stress hormones monitored in the animals. Moreover, the deck where the pigs were transported apparently affected only the norepinephrine levels, whilst the adrenaline and cortisol levels were not altered. Our results might help elaborate proposals and suggestions to minimise pig stress during transportation. To our knowledge, this is the first study evaluating pig transport stress responses using both plasma cortisol and catecholamine levels during exsanguination.

A higher level of stress was measured in the animals subjected to the 11 h-long transport, that included two rest stops without unloading of the animals. This result was expected and in line with the literature. Pigs transported for 6 h revealed a higher serum cortisol concentration than pigs transported for only 3 h [10]. Higher cortisol concentrations after longer transport times (5 h vs. 40 min) were also reported in other studies [28] and are likely owing to longer feed deprivation [29]. Moreover, the length of the journey could also cause injuries to the animal and, consequently, affect carcass quality and meat characteristics. For instance, the frequency of bruised carcasses increases with journey duration [30]. Other studies demonstrated that cortisol concentrations were considerably higher after short transport than after long transport and resting [31,32]. Other studies have observed a higher incidence of pale, soft and exudative (PSE) meat, as well as increased risk of fatigue and skin damage, in pigs subjected to a short-distance journey than in pigs transported for longer distances [33,34], although in those studies the transportation times were less than 2 h. It is clear that pigs transported for such a short distance do not have sufficient time to recover from the stress of loading in the truck. In fact, many authors [35,36] consider loading as a key factor for transport stress. The frequency of stress-related behaviours is greatest in the first hour of travel and tends to decrease with journey duration [37]. Warris et al. [38] reported that cortisol concentrations increased with loading and the first part of the journey, but then recovered as the journey continued. Nevertheless, with increasing transport duration, the stress level of transported animals grew again, correlated also with the prolonged time of feed and water deprivation [29].

In our study, we assessed the effects of journey duration on HPA-SAM activity. In detail, the increase of the three hormones was proportional to the journey distance, with the pigs travelling for 6.5 h displaying intermediate concentrations between those travelling for 3 h and 11 h. The different concentrations of the parameters analysed reflects the physiological response of an organism coping with a stressful situation. It is indeed not surprising that norepinephrine (later converted into epinephrine) was higher than epinephrine and cortisol, because this catecholamine, during restraint stress and during bleeding out, is released first from sympathetic nerve terminations and then from the adrenal medulla [39]. On the other hand, cortisol remains active in the body longer than either epinephrine or norepinephrine [39] and it is generally considered an indicator of physiological stress of an animal and its reaction to an environmental threat to its homeostasis [40]. It is worth noting that even if it is impossible to discriminate the effects of the journey from those of lairage and stunning, collecting blood at exsanguination is a common and non-invasive technique for the assessment of physiological responses of pigs and other animals to a journey toward a slaughterhouse [10,25,29]. This practice is indeed in line with the 3Rs code for conducting research using animals [41].

In agreement with previous observations, our findings highlight that, after transport, the interaction between the truck deck and the journey distance was not significant for cortisol levels [10]. Our results are however apparently in conflict with the conclusions drafted by Sommavilla et al. [32]. The latter authors showed that the interaction season × travel duration × compartment location influenced serum cortisol concentrations in an 18-h journey during summer, with cortisol values higher in pigs transported in the bottom deck compared with those located in the upper deck. However, the blood cortisol level increase observed in that study [32] could be interpreted as a response to heat stress. In fact, during winter, the temperature is lower in the top compartments, while during summer it is higher in the bottom rear compartment [32]. Accordingly, the extreme environmental temperature during summer and winter likely influenced those results. Our study was conducted during spring, with a mild climate, and this likely explained the fact that there was no influence of temperature and humidity on the pigs’ cortisol levels in our study. Whilst the truck deck level was not significant for the epinephrine and cortisol levels observed in our study, it was significant for the norepinephrine levels. In particular, norepinephrine showed higher levels in the pigs transported in the upper deck than in the pigs loaded in the bottom deck, whilst pig transported in the middle deck showed intermediate values. This increase of norepinephrine levels could be explained considering that animals transported on the third deck could have suffered from more stress than those transported on the other levels. Indeed, these pigs are obliged to use internal ramps at loading and unloading, as also suggested in the literature [8]. Protracted norepinephrine release is observed in the case of traumatic injury and this may be attributed mainly to peripheral stimulation [42]. It is possible that norepinephrine was released from the damaged tissue and this may explain its high plasma concentration in the pigs of the third deck. Moreover, pigs located in the front and rear compartments or in the upper and lower decks have an increased number of carcass skin bruises and a reduced pork quality [43]. A tendency towards higher skin damage scores was found in pigs transported on the trailer compared with those located on the lorry because the trailer is subjected to more vibrations and movements than the lorry [9]. The fact that we only analysed samples of pigs travelling in the trailer may have therefore affected our results to some extent. It has been reported that epinephrine is not useful as a biomarker of stress because it is best measured 5 min after stimuli; on the other hand, norepinephrine exhibits a more protracted response and it is an indicator of tissue damage [42]. Consequently, assessing norepinephrine at bleeding may become a useful indirect indicator of carcass and meat quality.

In compliance with Annex III of Council Regulation (EC) No. 1099/2009 [23], according to which animals should be unloaded as quickly as possible after arrival and subsequently slaughtered without undue delay, the pigs of our study were immediately conducted inside the slaughterhouse for the stunning phase, without a resting period. This situation could have contributed to the higher cortisol levels recorded in our study at exsanguination than those documented after a recovery time spent in the lairage facilities [10,44]. Warriss et al. [39] demonstrated that cortisol levels were significantly higher in pigs slaughtered on arrival at the abattoir compared with pigs held in the lairage for 3 h before slaughter. Holding pigs for 3 h in the lairage before slaughter helped the stress parameters to return to the baseline and pigs to rest and recover, by influencing their behaviour and handling. In terms of pork quality, optimal lairage times are from 1 to 3 h [45]. Indeed, a resting period of <30 min resulted in the highest incidence of pale and soft/exudative loins, while a 3 h rest significantly improved quality by reducing this phenomenon [46,47]. It is clear that a resting period of about 3 h provides consistent improvements in animal welfare and, consequently, in meat quality, with economic benefits for the slaughterhouse. In fact, high levels of plasma cortisol at slaughter are associated with higher pH at 3, 6 and 24 h post stunning, lower lightness and redness of meat and lower drip loss [48]. Considering that elevated levels of catecholamines and glucocorticoids stimulate hepatic glycogenolysis, leading to a reduction of post-mortem lactic acid and an unacceptable conversion from muscle to meat [40,49], the animals should rest after unloading from the truck. Transport distance alone does not seem to determine high stress levels in pigs and transport-derived stress may be reduced by increasing the time spent in lairage before stunning [50]. In fact, lairage rest allows animals to recover from loading, transport and unloading stress. Grandin [51] also recommended that pigs should rest 2 to 4 h before entering the stunning procedure. Based on our preliminary results, at least the pigs transported for 11 h, in which the highest levels of the hormones were identified, should rest for a few hours before stunning. This practice might help the animals recover, resulting in better carcass quality.

The 11 h-long journey was carried out in a vehicle approved for short journeys and this had negative effects on pigs’ welfare. From this perspective, even if Council Regulation (EC) No. 1/2005 [7] defines as a “long journey” only journeys that exceed 8 h, the same Regulation reports that the Member States may grant derogations for means of transport by road for journeys not exceeding 12 h to reach the final destination. Consequently, journeys not exceeding 12 h are made with trucks without the additional provisions required for long journeys. In particular, those journeys are carried out without a temperature monitoring and recording system, without sensors located in the parts of the lorry which are most likely to experience the worst climatic conditions, without a warning system able to alert the driver when the temperature exceed the maximum or the minimum limits and, finally, without an appropriate navigation system allowing for recording and providing information equivalent to those mentioned in the journey log. Therefore, it seems that Council Regulation (EC) No. 1/2005 [7] is inadequate when extending a national 8 h journey by an additional 4 h, as a 12 h journey should not be considered short for the animals. Indeed, without these additional requirements for long journeys, it is difficult to ensure a constant environment during the transport of animals, regardless of the daytime, season, position of the animals, deck loading and other possible variables.

Other factors such as prolonged time spent in the lairage facilities could minimise the adverse impacts of transport-stress on pigs’ health and welfare. A standardised method on how to conduct pigs handling after unloading should be implemented among European countries to safeguard animal welfare and meat quality. Indeed, there is a legislative gap in terms of animal handling after transportation. Council Regulation (EC) No. 1099/2009 [23] indicates that animals should be unloaded as quickly as possible after arrival and subsequently slaughtered without undue delay, thus failing to protect pigs transported for more than 8 h. These animals would need more time to recover from fatigue and stress owing to a longer journey.

Our results need to be interpreted with caution because our pilot study was limited by several factors. First of all, the tested animals were transported only in the trailer. We chose these animals for a logistical reason because they are usually the first to be unloaded. Accordingly, our results may be valid only for the animals transported in the trailer and cannot be extended to pigs transported in the lorry or another type of vehicle. Secondly, the pigs sampled pre and post transport were not the same because the animals were randomly selected during exsanguination in order to avoid a slowdown in pig slaughter times. However, they belonged to the same breeding group and the slaughterhouse requires that all farms raise animals in the same way, with the same modalities and with a food regimen quite comparable. Thirdly, we tested only a journey for each distance, so our data need to be repeated more times and using the same animals pre and post transport. Fourthly, stress responses were documented using only endocrine parameters because other stress indicators (pig behaviour during transport, rectal temperature, heart rate, haematological and biochemical analytes) could not be evaluated. Moreover, it was impossible to evaluate if catecholamine and cortisol values were related to journey, lairage or stunning because these three phases were not separated. The lairage and the stunning steps were the same for all animals. However, considering that the pigs of the upper deck were the last to be unloaded and that exsanguination took place in order of unloading, the deck may have biased the lairage duration. Another limit of our study was the failure to register temperature and humidity data on the truck. In compliance with the current European Regulation [7], recording of environmental parameters is mandatory only in the case of long journeys. Additionally, we were not allowed to put instrumentations inside the truck. Finally, the lack of information regarding carcass bruises and injuries, as well as pH and drip loss, made it impossible for us to analyse changes in meat quality related to the duration of the journey and the deck. Regardless of these limitations, this study reports endocrine changes in pigs, thus increasing our knowledge about transport stress in pigs and their catecholamine levels.

## 5. Conclusions

This pilot study of three commercial truckloads confirmed that journey duration is a major risk factor for transport-derived stress in pigs. Our results also suggest that pigs transported in the higher deck of the truck may have a higher level of norepinephrine at exsanguination, which may be suggested as an indirect indicator of carcass quality. These preliminary results need to be confirmed by future studies, using a larger sample size and evaluating additional welfare indicators to assess if and how they differ with respect to the journey duration, the deck and the time spent in the lairage facilities.

## Figures and Tables

**Table 1:** Estimated Least Squares Means (LSMs) and Standard Errors (SE) of the levels of cortisol (nmol/L), epinephrine (ng/L) and norepinephrine (ng/L) in plasma collected before (T0) and after (T1) a journey from three different farms towards the same slaughterhouse. LSMs within the row bearing different superscripts (A, B, C) are significantly different for *p* ≤ 0.01. LSMs within the tested hormones and in the same column followed by different superscripts (E, F) are significantly different for *p* ≤ 0.01.

Parameters	Time	J1 (11 h)	J2 (6.5 h)	J3 (3 h)	SE	Time*Farm *p*-Value
		A	B	C		
Cortisol (nmol/L)	T0	36 ^E^	36 ^E^	38 ^E^	5.03	<0.0001
	T1	139 ^A,F^	118 ^B,F^	94 ^C,F^		
Epinephrine (ng/L)	T0	867 ^E^	873 ^E^	765 ^E^	435	<0.0001
	T1	19,414 ^A,F^	13,735 ^B,F^	9232 ^C,F^		
Norepinephrine (ng/L)	T0	434 ^E^	440 ^E^	384 ^E^	396	<0.0001
	T1	17,469 ^A,F^	13,417 ^B,F^	6227 ^C,F^		

The deck-journey distance interaction was not significant on epinephrine, norepinephrine or cortisol levels collected at arrival (T1). There was a significant effect of deck level on norepinephrine levels (*p* < 0.0001), a tendency to influence epinephrine levels (*p* = 0.073) but no effect on cortisol levels (*p* = 0.945; Table 2).

**Table 2:** Estimated Least Squares Means (LSMs) and Standard Errors (SE) of the levels of cortisol (nmol/L), epinephrine (ng/L) and norepinephrine (ng/L) collected during the exsanguination phase in pigs transported in different truck decks (1, 2, 3). LSMs within the row bearing different superscripts (A, B) are significantly different for *p* ≤ 0.01.

Parameters	Truck Decks			SE	*p*-Value
	1	2	3		
Cortisol (nmol/L)	117	118	116	5.9	0.945
Epinephrine (ng/L)	14,910	13,522	13,950	880	0.073
Norepinephrine (ng/L)	10,910 ^A^	12,790 ^B^	13,405 ^B^	340	<0.0001

## References

[1] Xiong Y., Green A.R., Gates R. (2015). Characteristics of Trailer Thermal Environment during Commercial Swine Transport Managed under U.S. Industry Guidelines. Animals.

[2] Gȩbska M. (2017). Legal and organizational aspects of the transport of pigs in Poland and selected EU countries. Changes in the Live Pig Market in Different Countries.

[3] Bench C., Schaefer A.L., Faucitano L., Faucitano L., Schaefer A.L. (2008). The welfare of pigs during transport. The Welfare of Pigs-From Birth to Slaughter.

[4] Brown J.A., Samarakone T.S., Crowe T., Bergeron R., Widowski T., Correa J.A., Faucitano L., Torrey S., Gonyou H.W. (2011). Temperature and Humidity Conditions in Trucks Transporting Pigs in Two Seasons in Eastern and Western Canada. Trans. ASABE.

[5] Costa O.D., Faucitano L., Coldebella A., Ludke J., Peloso J., Roza D.D., Da Costa M.J.R.P. (2007). Effects of the season of the year, truck type and location on truck on skin bruises and meat quality in pigs. Livest. Sci..

[6] Padalino B., Raidal S.R. (2020). Effects of Transport Conditions on Behavioural and Physiological Responses of Horses. Animals.

[7] European Commission (EC) (2005). Council Regulation (EC) 1/2005 of 22 December 2004 on the protection of animals during transport and related operations and amending Directives 64/432/EEC and 93/119/EC and Regulation (EC) No 1255/97. O. J. Eur. Union..

[8] Correa J.A., Gonyou H., Torrey S., Widowski T., Bergeron R., Crowe T., Laforest J.-P., Faucitano L. (2014). Welfare of Pigs Being Transported over Long Distances Using a Pot-Belly Trailer during Winter and Summer. Animals.

[9] Arduini A., Redaelli V., Luzi F., Dall’Olio S., Pace V., Costa L.N. (2017). Relationship between Deck Level, Body Surface Temperature and Carcass Damages in Italian Heavy Pigs after Short Journeys at Different Unloading Environmental Conditions. Animals.

[10] Newman D., Young J.M., Carr C., Ryan M., Berg E.P. (2014). Effect of Season, Transport Length, Deck Location, and Lairage Length on Pork Quality and Blood Cortisol Concentrations of Market Hogs. Animals.

[11] Schaefer F., Chen Y., Tsao T., Nouri P., Rabkin R. (2001). Impaired JAK-STAT signal transduction contributes to growth hormone resistance in chronic uremia. J. Clin. Investig..

[12] Dallman M.F., Hellhammer D., Contrada R.J., Baum A. (2011). Regulation of the hypothalamo-pituitaryadrenal axis, chronic stress, and energy: The role of the brain and networks. The Handbook of Stress Science: Biology, Psychology, and Health.

[13] Gayrard V., Alvinerie M., Toutain P. (1996). Interspecies variations of corticosteroid-binding globulin parameters. Domest. Anim. Endocrinol..

[14] Manteca X., Manteca X. (2009). Conceptos generales de bienestar animal. Etología Veterinaria.

[15] Hambrecht E., Eissen J.J., Nooijen R.I.J., Ducro B.J., Smits C.H.M., Hartog L.A.D., Verstegen M.W.A. (2004). Preslaughter stress and muscle energy largely determine pork quality at two commercial processing plants. J. Anim. Sci..

[16] Foury A., Devillers T., Sanchez M.P., Griffon H., Le Roy P., Mormede P. (2005). Stress hormones, carcass composition and meat quality in Large White X Duroc pigs. Meat Sci..

[17] Hambrecht E., Eissen J.J., Newman D.J., Smits C.H.M., Hartog L.A.D., Verstegen M.W.A. (2005). Negative effects of stress immediately before slaughter on pork quality are aggravated by suboptimal transport and lairage conditions. J. Anim. Sci..

[18] Dokmanovic M., Baltic M.Z., Duric J., Ivanovic J., Popovic L., Todorovic M., Markovic R., Pantic S. (2015). Correlations among Stress Parameters, Meat and Carcass Quality Parameters in Pigs. Asian-Australas. J. Anim. Sci..

[19] A Geverink N., De Jong I.C., Lambooij E., Blokhuis H., Wiegant V.M. (1999). Influence of housing conditions on responses of pigs to preslaughter treatment and consequences for meat quality. Can. J. Anim. Sci..

[20] National Research Council (2012). Nutrient Requirements of Swine: Eleventh Revised Edition.

[21] European Economic Community (EEC) (1985). Council Regulation (EEC) 3820/1985 of 20 December 1985 on the harmonization of certain social legislation relating to road transport. O. J. Eur. Communities.

[22] Edwards L.N., Grandin T., Engle T., Ritter M.J., Sosnicki A., Carlson B., Anderson D.B. (2010). The effects of pre-slaughter pig management from the farm to the processing plant on pork quality. Meat Sci..

[23] European Commission (EC) (2009). Council Regulation (EC) 1099/2009 of 24 September 2009 on the protection of animals at the time of killing. O. J. Eur. Union..

[24] Ceci E., Marchetti P., Samoilis G., Sportelli S., Roma R., Barrasso R., Tantillo G., Bozzo G. (2017). Determination of plasmatic cortisol for evaluation of animal welfare during slaughter. Ital. J. Food Saf..

[25] Bozzo G., Barrasso R., Marchetti P., Roma R., Samoilis G., Tantillo G.M., Ceci E. (2018). Analysis of Stress Indicators for Evaluation of Animal Welfare and Meat Quality in Traditional and Jewish Slaughtering. Animals.

[26] The R Project for Statistical Computing. http://www.r-project.org/.

[27] Fox J., Weisberg S. (2019). An R Companion to Applied Regression.

[28] Chai J., Xiong Q., Zhang C., Miao W., Li F., Zheng R., Peng J., Jiang S. (2010). Effect of pre-slaughter transport plant on blood constituents and meat quality in halothane genotype of NN Large White×Landrace pigs. Livest. Sci..

[29] Toscano M.J., Lay D.C., Craig B.A., Pajor E.A. (2007). Assessing the adaptation of swine to fifty-seven hours of feed deprivation in terms of behavioural and physiological responses. J. Anim. Sci..

[30] Mota-Rojas D., Becerril M., Lemus C., Sanchez P., Gonzalez M., Olmos S., Ramírez R., Alonso-Spilsbury M. (2006). Effects of mid-summer transport duration on pre- and post-slaughter performance and pork quality in Mexico. Meat Sci..

[31] Tateo A., Padalino B., Boccaccio M., Maggiolino A., Centoducati P. (2012). Transport stress in horses: Effects of two different distances. J. Veter. Behav..

[32] Sommavilla R., Faucitano L., Gonyou H., Seddon Y., Bergeron R., Widowski T., Crowe T., Connor L.M., Scheeren M.B., Goumon S. (2017). Season, Transport Duration and Trailer Compartment Effects on Blood Stress Indicators in Pigs: Relationship to Environmental, Behavioral and Other Physiological Factors, and Pork Quality Traits. Animals.

[33] Gajana C., Nkukwana T., Marume U., Muchenje V. (2013). Effects of transportation time, distance, stocking density, temperature and lairage time on incidences of pale soft exudative (PSE) and the physico-chemical characteristics of pork. Meat Sci..

[34] Guardia M., Estany J., Balasch S., Oliver M., Gispert M., Diestre A. (2005). Risk assessment of DFD meat due to pre-slaughter conditions in pigs. Meat Sci..

[35] Grandin T. (1999). Easy tips for low stress cattle handling. Large Anim. Pract..

[36] Wikner I., Gebresenbet G., Nilsson C. (2003). Assessment of air quality in a commercial cattle transport vehicle in Swedish summer and winter conditions. DTW Dtsch. Tierarztl. Wochenschr..

[37] Padalino B., Raidal S.R., Knight P.K., Celi P., Jeffcott L., Muscatello G. (2018). Behaviour during transportation predicts stress response and lower airway contamination in horses. PLoS ONE.

[38] Warriss P., Brown S., Knowles T., Kestin S., Edwards J., Dolan S., Phillips A. (1995). Effects on cattle of transport by road for up to 15 hours. Veter. Rec..

[39] Gregory N.G. (1998). Physiology of Stress, Distress, Stunning and Slaughter. Animal Welfare and Meat Science.

[40] Knowles T.G., Warriss P.D., Grandin T. (2000). Stress physiology of animals during transport. Livestock Handling and Transport.

[41] National Health and Medical Research Council (2013). Australian Code for the Care and Use of Animals for Scientific Purposes.

[42] Mellor D.J., Stafford K.J., E Todd S., E Lowe T., Gregory N.G., A Bruce R., Ward R.N. (2002). A comparison of catecholamine and cortisol responses of young lambs and calves to painful husbandry procedures. Aust. Veter. J..

[43] Scheeren M.B., Gonyou H.W., Brown J., Weschenfelder A.V., Faucitano L. (2014). Effects of transport time and location within truck on skin bruises and meat quality of market weight pigs in two seasons. Can. J. Anim. Sci..

[44] Faucitano L., Goumon S. (2018). Transport of pigs to slaughter and associated handling. Advances in Pig Welfare.

[45] Warriss P.D. (2003). Optimal lairage times and conditions for slaughter pigs: A review. Veter. Rec..

[46] Milligan S.D., Ramsey C.B., Miller M.F., Kaster C.S., Thompson L.D. (1998). Resting of pigs and hot-fat trimming and accelerated chilling of carcasses to improve pork quality. J. Anim. Sci..

[47] Fortin A. (2002). The effect of transport time from the assembly yard to the abattoir and resting time at the abattoir on pork quality. Can. J. Anim. Sci..

[48] D’Eath R.B., Turner S.P., Kurt E., Evans G., Thölking L., Looft H., Wimmers K., Murani E., Klont R., Foury A. (2009). Pigs’ aggressive temperament affects pre-slaughter mixing aggression, stress and meat quality. Animals.

[49] Onenc A., Kaya A. (2004). The effects of electrical stunning and percussive captive bolt stunning on meat quality of cattle processed by Turkish slaughter procedures. Meat Sci..

[50] Martoccia L., Brambilla G., Macrì A., Moccia G., Cosentino E. (1995). The effect of transport on some metabolic parameters and meat quality in pigs. Meat Sci..

[51] Grandin T. (1994). Farm animal welfare during handling, transport, and slaughter. J. Am. Veter. Med. Assoc..

